# Neuro-cognitive assessment of intentional control methods for a soft elbow exosuit using error-related potentials

**DOI:** 10.1186/s12984-022-01098-0

**Published:** 2022-11-11

**Authors:** Nicholas Tacca, John Nassour, Stefan K. Ehrlich, Nicolas Berberich, Gordon Cheng

**Affiliations:** Institute for Cognitive Systems, Technical University of Munich, Munich, Germany

**Keywords:** Soft robotics, Exosuit, EEG, EMG, Human–machine interface

## Abstract

Soft exosuits offer promise to support users in everyday workload tasks by providing assistance. However, acceptance of such systems remains low due to the difficulty of control compared with rigid mechatronic systems. Recently, there has been progress in developing control schemes for soft exosuits that move in line with user intentions. While initial results have demonstrated sufficient device performance, the assessment of user experience via the cognitive response has yet to be evaluated. To address this, we propose a soft pneumatic elbow exosuit designed based on our previous work to provide assistance in line with user expectations utilizing two existing state-of-the-art control methods consisting of a gravity compensation and myoprocessor based on muscle activation. A user experience study was conducted to assess whether the device moves naturally with user expectations and the potential for device acceptance by determining when the exosuit violated user expectations through the neuro-cognitive and motor response. Brain activity from electroencephalography (EEG) data revealed that subjects elicited error-related potentials (ErrPs) in response to unexpected exosuit actions, which were decodable across both control schemes with an average accuracy of 76.63 ± 1.73% across subjects. Additionally, unexpected exosuit actions were further decoded via the motor response from electromyography (EMG) and kinematic data with a grand average accuracy of 68.73 ± 6.83% and 77.52 ± 3.79% respectively. This work demonstrates the validation of existing state-of-the-art control schemes for soft wearable exosuits through the proposed soft pneumatic elbow exosuit. We demonstrate the feasibility of assessing device performance with respect to the cognitive response through decoding when the device violates user expectations in order to help understand and promote device acceptance.

## Background

Assistive technologies offer promise to augment human capabilities in order to provide assistance to reduce human energy expenditure [1]. For able bodied people, assistive technologies, such as exoskeletons, can mitigate muscle fatigue and metabolic cost by providing assistance that works in parallel with the human body [2–7]. Musculoskeletal injuries due to over exertion and repetitive tasks are one of the leading work-related health problems [8]. Exoskeletons that operate in conjunction with the human body may help reduce injuries by promoting proper lifting techniques and reducing the overall physical human load [9].

Despite practical exoskeleton testing in manufacturing settings, such as the automotive industry, as well as few non-manufacturing domains, large-scale usage adoption beyond experimental use have been limited compared with societal expectations [10]. An important factor for device adoption is the synergistic interface between human and machine [11, 12]. Rigid exoskeletons have had success supporting gait or providing upper body assistance [13, 14]. The physical interface, however, is limited due to the rigidity and weight creating a low force to weight ratio and limited portability [15]. Solutions that conform to the human body and move naturally with human actions are important for continued use. Soft materials can provide a comfortable and lightweight interface that do not restrict movement [2]. The soft interface can act as an external layer that works in parallel with muscles to support human joint mechanics [5]. This provides a significant advantage over rigid devices for long-term use and adoption in a usable form-factor.

Despite potential benefits for end users, its widespread adoption has been thwarted due to the control challenges based on the non-linear response of soft materials, difficulty in state estimation, and reduced assistance magnitude when compared to rigid devices [16]. Recent advances in the control and development of soft exosuits have demonstrated the importance of decoding user motor intentions for control [6]. Assistive devices should decode human motor intentions and provide assistance in accordance with user expectations to work seamlessly with the wearer. An intuitive control system and overall positive user-experience are necessary for continued usage [11]. Users should ideally experience a sense of embodiment such that the device operates as an extension of their body. Embodiment, in this context, can be defined as the incorporation of artificial body parts or extensions of the body into the user’s own body schema that are perceived as part of their own being [17].

Various methods of active exoskeleton control have been proposed to enhance embodiment and user-experience, which range in the level of user involvement versus shared or automatic control of the device [18]. At the very basic level, a simple user-control method based on trigger activation may suffice in repetitive industrial applications or rehabilitation training. By taking advantage of human-in-the-loop control schemes, users can directly activate assistance when needed. While this promotes flexibility, it also limits function and enforces users to alter their normal behavior to compensate for the additional assistance tool. This leads to a non-intuitive interaction between the user and device, since additional mental workload is required to operate the device [6, 19].

For these reasons many researchers have proposed automatic support control systems to be more applicable for use in daily life [3–5, 20–26]. Through a kinematic mapping of the body, it is possible to compensate joints based on limb position and movement. Typically, a robotics-based gravity compensation scheme is deployed whereby a reference torque due to gravity is calculated and the exoskeleton provides equal and opposite torque to account for the mass due to gravity [27]. An assumption of the exoskeleton mass and wearer is needed in order to provide adequate torque assistance. Previous works demonstrate, however, that a gravity compensation control scheme provides a suitable and simple approach to assist the wearer even when interacting with different object masses that significantly reduces muscle activity [3, 4, 6, 22, 25]. In this setup, the user must learn to compensate for the same level of assistance when requiring more or less support depending on the interaction. Additionally, a kinematic model can provide high-level information of the task. By understanding whole body activities through a taxonomy of movements and postures such as standing, bending, walking, and other goal-oriented tasks, assistance can be provided based on the activity [28]. While this information may provide a better understanding of task-level constraints, it lacks a full understanding of how much assistance to provide for the select actions defined through the movement taxonomy.

To address some of the limitations of only using a kinematic model to infer intention, several previous works have proposed neural-based control of exoskeletons via muscle activation measured from electromyography (EMG) signals [5, 20, 21, 23, 24, 29–32]. Advantages of using EMG is that the activation occurs before movement onset, allowing the controller to predict intended motion prior to executing the action. Researchers have utilized muscle models (e.g. the Hill Model [33, 34]) to estimate muscle force based on muscle activity [5, 20, 21]. With this assumption of muscle force, a subsequent torque about the joint can be determined through which a compensating assistance torque can be provided to the user. This allows the user to interact with a variety of different object masses with the exoskeleton able to continuously adapt to provide assistance according to the level of muscle activity required to lift the object. When comparing muscle-based control schemes with a traditional gravity compensation approach, both have shown to provide assistance in accordance with user intentions while minimizing muscle activity [6]. However, muscle-based approaches have shown to be more versatile, able to adapt in dynamic conditions. On the other hand, muscle-based approaches require additional EMG sensors that need to be placed directly on the skin and typically involve some sort of calibration procedure. Therefore, depending on the application, a gravity compensation control scheme may be sufficient.

While previous groups have demonstrated sufficient device performance based on reduced muscle activity, the cognitive response of human-interaction with these devices has been neglected. Human–machine interaction studies have demonstrated the ability to decode error-related potentials (ErrPs) from electroencephalography (EEG) signals, indicative of events in which a robot violates the human’s expectations. ErrPs are a type of event-related potential (ERP) characterized by an electrophysiological response to internal or external events in performance monitoring related to response conflict [35]. The ability to decode ErrPs can provide a level of understanding of the human’s perceived mistake made by a machine and update its response to align future actions with human preferences [36–41]. While these studies focus on the human interaction with an autonomous robot, it is likely the same principles can be applied to a wearable robotic device. In this case, it is imperative for the robot and the human to move synchronously since they are physically connected. If the human initiates an action, then the device must follow the intended action. Conversely, if the device initiates the movement, then the human must perform the action independent of the user’s desired intentions. With this direct coupling, an understanding of when the exoskeleton violates expectations based on neuro-cognitive measures from EEG signals can elucidate moments in which the exoskeleton fails to decode the user’s intentions.

We propose a soft pneumatic elbow exosuit designed based on our previous work [7] to provide active elbow assistance support. Intentions are decoded through a gravity compensation and muscle-based approach to provide assistance in accordance with user expectations. We compare intentional control strategies using the soft pneumatic elbow exosuit, similar comparison has been carried out on a tendon-driven actuation [6]. In addition, we take a human-centered approach in an attempt to understand the user-experience with the exoskeleton through a neuro-cognitive assessment evaluating different intentional control schemes. We investigate the feasibility of detecting when the exoskeleton violates user expectations through the neuro-cognitive and motor response in an effort to enhance the cognitive interaction with the device.

The aim of this work, therefore, is to assess the proposed exosuit performance with respect to the user’s cognitive and motor response by decoding when the device fails to predict intentions by violating user expectations. With this objective in mind, the paper is structured with the methods describing the proposed exosuit design and control schemes. A neuro-cognitive assessment follows the control system methods aimed at assessing the control solutions and neuro-cognitive response. The main contributions of our work consist of the following:

**Primary**

1. A neuro-cognitive assessment to determine the feasibility of decoding instances in which the the proposed exosuit violated user expectations based on neuro-cognitive measures and the motor response.

**Secondary**

1. Design of a soft pneumatic exosuit based on our previous work [7] capable of providing elbow flexion assistance.
2. Development of a high-level control system based on existing state-of-the-art soft exosuit control methods using gravity compensation and muscle activation optimized with a low-level PID controller.

## Methods

### Soft pneumatic elbow exoskeleton system design

**Fig. 1:**
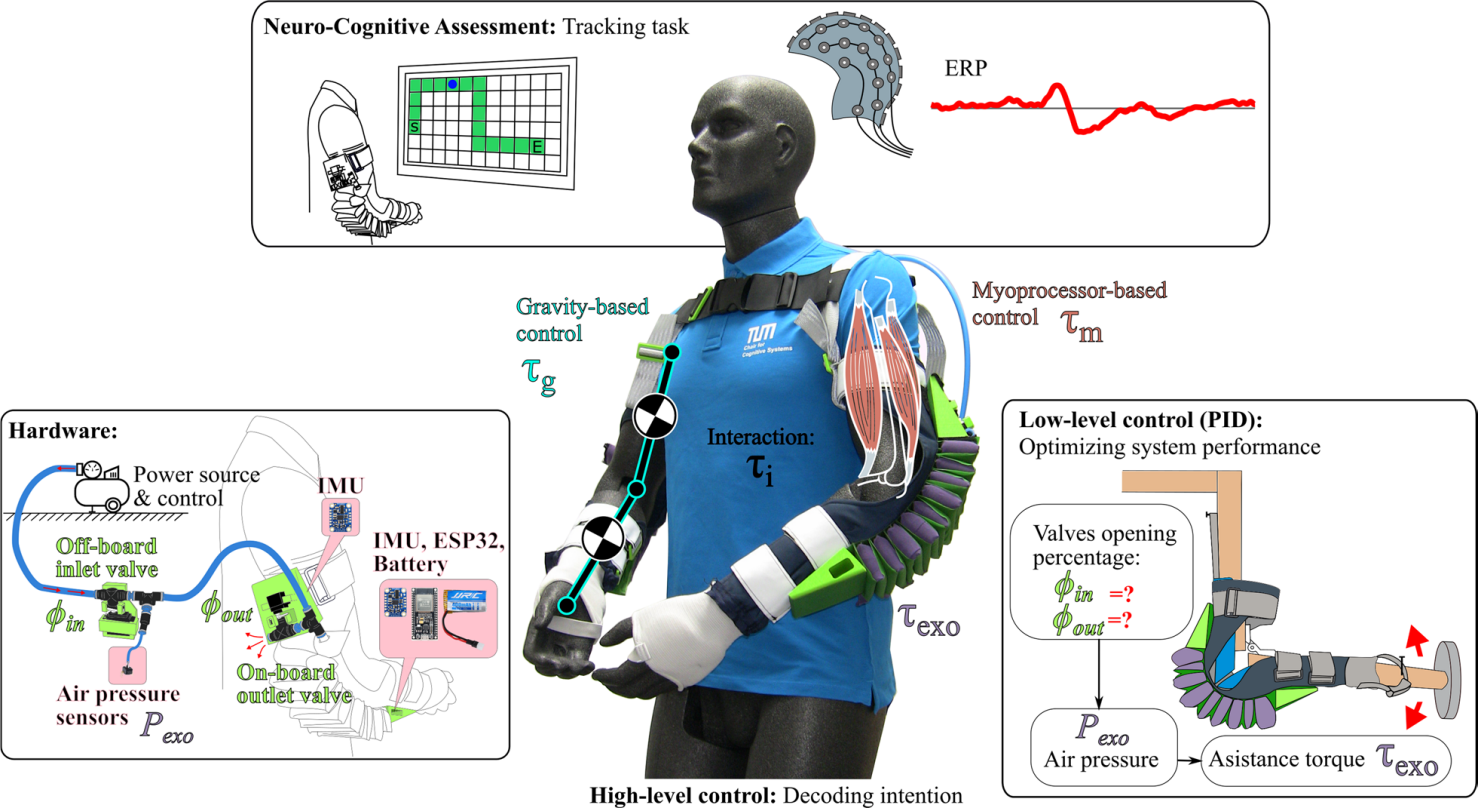
*System and experiment methods overview. Hardware.* The soft pneumatic exosuit consists of two independent elbow sleeves with actuator tubes that snake through the posterior portion of the sleeves to provide flexion assistance. An air compressor power source is used to inflate the exosuit. Inlet valves and pressure sensors are off-board housed within a separate control box. Outlet valves are located directly on the upper arm at the inlet to the actuator. Inlet and outlet valve opening percentages $\phi _{in}$ and $\phi _{out}$ are modulated to control exosuit pressure. Two IMUs per side, ESP32 microcontroller, and battery are embedded in the sleeve to determine exosuit kinematics. *High-level control: Decoding intention.* A decoded required torque $\tau _r$ is determined via a gravity compensation scheme ($\tau _g$) or a myoprocessor scheme ($\tau _m$) based on muscle activity measured with supplementary Delsys EMG sensors. exosuit torque $\tau _{exo}$ being supplied to the wearer is linearly dependent on the actuator tube air pressure. The interaction torque $\tau _i$ between the exosuit torque provided and decoded torque required from the user should be minimized as much as possible. *Low-level control: Optimizing system performance.* A PID controller was used to modulate valve opening percentages $\phi _{in}$ and $\phi _{out}$ to minimize the interaction torque $\tau _i$. *Neuro-Cognitive Assessment: Tracking task.* An EEG study was conducted to determine when the exosuit violated user expectations from the ERP in response to control errors to understand and enhance the cognitive interface with the exosuit

The soft exosuit system consists of two independent elbow sleeves connected with a shoulder strap and pad to provide elbow support and a control box consisting of inlet valves and pressure sensors (Fig. 1). A snaking tube weaves through the posterior portion of the sleeve so that when pressurized, it provides flexion assistance. In contrast to our previous design [7, 42], the current exosuit has been adapted to incorporate sensors for intention detection, as well as onboard valve-actuation for dynamic control.

#### Sensor integration

A single IMU module is placed within the 3D printed housing on the forearm per each side of the exosuit with two modules in total (Fig. 1). Each module is wired to two 9-axis Adafruit BNO055 absolute orientation sensors and contain a ESP-WROOM-32 system on a chip microcontroller, and LiPo battery (3.7 V). The IMU sensors are located in the shin-pad brace on the forearm and upper arm on both sides of the exosuit. The absolute orientation in the form of the quaternion is processed at a rate of 100 Hz from the forearm module, which communicates via Bluetooth serial port to the software system. This sampling frequency limits the refresh rate of the overall closed loop system. The IMU sensors are used to determine the relative orientation in space to get a kinematic model of the upper body in order to infer desired human motion. A predefined calibration position was used to determine the mapping of joint positions of average body limb segment lengths based on average male and female anthroprometric dimensions [43].

The Delsys Trigno EMG system can be seamlessly incorporated with the exosuit system to obtain high quality EMG signals. For all tests using EMG, two electrodes from the Delsys Trigno QUATTRO sensors were positioned on the biceps and triceps according to SENIAM [44] guidelines. The overall hardware of the Delsys system is independent from the current exosuit system and is only required when using the muscle-based control option.

Two pressure sensors are contained within the exosuit control box in series with the inlet air flow into the suit (Fig. 1). They are connected to an ESP-WROOM-32 system on a chip microcontroller housed within the control box. Each pressure sensor measures the pressure within the tube on the left and right side of the exosuit respectively. Monitoring the pressure ensures the device operates within a safe limit, as well as an estimate of the exosuit assistance torque that is being supplied to the wearer.

#### Pneumatic actuation system

Air flows into the system via a 6mm diameter tube directly connected to an air compressor. The system has the potential to be fully mobile by incorporating a high-pressure air (HPA) tank in place of the air compressor. A safety relief valve is incorporated directly outside the control box to limit the air pressure in the system between 1.5 and 3 bar. Additionally, a low-pressure regulator is used to modulate the input air pressure from the air compressor. The inlet air pressure for device operation was set to 3 bar for all experiments as this provides a suitable system response while minimizing high pressure on the actuator tubes.

The exosuit system contains a valve-actuation sub-system with one inlet and one outlet valve per side of the exosuit (Fig. 1). Both inlet valves are located within the control box between the air compressor and the exosuit tube. Each valve is controlled independently with a servomotor. The two servomotors are connected to the same microcontroller housed in the control box that reads exosuit pressure. The microcontroller communicates with the software system via serial port to control the valve diameter by adjusting the servomotor angle. Outlet valves are located directly on each side of the exosuit near the exosuit tube inlet on the upper arm so that air can be rapidly released when needed. Each outlet valve is connected to the microcontroller and controlled via a servomotor in the same way as the inlet valves. Based on the level of assistance needed, the inlet and outlet valves modulate the amount of air flowing into and out of the system.

#### Software system

All sensor information and actuator signals are integrated using the open-source Robotics Operating System (ROS) Kinetic to allow for real-time communication between nodes for exosuit control. Nodes are Python 3 based and run on the Ubuntu 16.04 operating system with the option to become fully mobile in the future. Due to the modularity of ROS, the exosuit can be operated with different control schemes sequentially or in parallel with both arms using a different control method. This provides the option to directly compare control schemes in real-time and then update control based on preferences.

### Control system

**Fig. 2:**
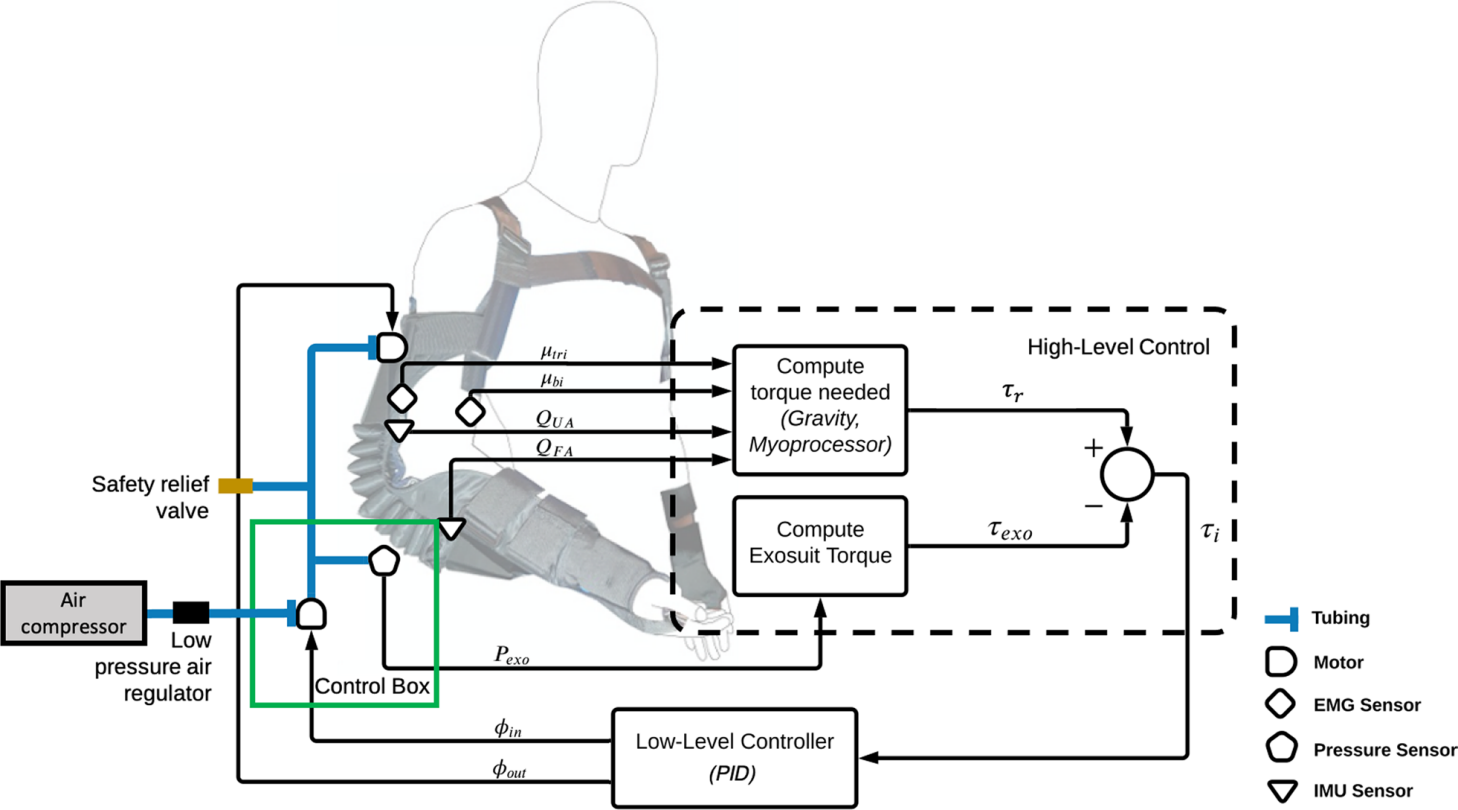
*Control system for the soft pneumatic elbow exosuit.* $\mu _{tri}$ and $\mu _{bi}$ indicate muscle activations determined from the Delsys Trigno EMG system in the triceps and biceps muscles respectively. $Q_{UA}$ and $Q_{FA}$ indicate the quaternion data collected from the IMU sensors for the upper arm and forearm respectively. $P_{exo}$ is the exosuit pressure reading from the pressure sensor housed within the control box. These sensor readings feed into the high-level control scheme that determines the required torque $\tau _r$ (gravity: $\tau _g$ or myoprocessor: $\tau _m$), as well as the exosuit torque $\tau _{exo}$. The output of the high-level controller is an interaction torque $\tau _i$ that acts as an input to the low-level controller. At this stage, the low-level controller aims to minimize the interaction between the user and exosuit assistance by minimizing the interaction torque via a PID controller. The output of the low-level controller is the opening percentages of the inlet valve $\phi _{in}$ and outlet valve $\phi _{out}$ respectively which get relayed to the servomotors controlling the inlet and outlet valves for air flow

Two control schemes based on existing methods were implemented to evaluate and validate performance with the proposed exosuit construction, as well as to enhance ease-of-use for an intuitive user experience. High level control schemes that aim to predict user intentions include gravity compensation and myoprocessor control (Fig. 2). A low-level PID controller was used to optimize the system response to the decoded intention.

#### High-level control: decoding user intention

**Fig. 3:**
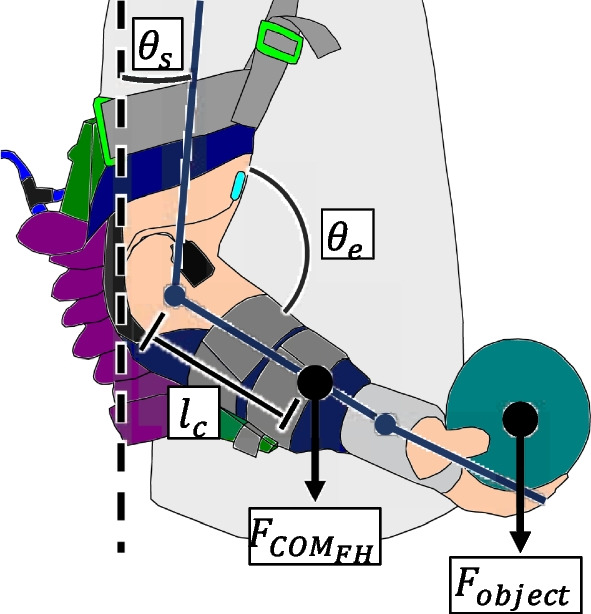
*Schematic of arm model with soft exosuit.* The force due to the forearm and hand combined center of mass ($F_{COM_{FH}}$) and the force due to the mass of a held object ($F_{object}$) determine the required compensation torque. The perpendicular distance between the elbow joint to the center of mass $COM_{FH}$ is denoted as $l_c$. The shoulder angle and elbow angle are represented by $\theta _s$ and $\theta _e$ respectively

*Gravity compensation control* An automatic support detection based on a gravity compensation was employed for continuous user control of the exosuit. The level of assistance required is inferred based on the torque about the elbow due to gravity assuming the shoulder angle $\theta _s$ is 0° with respect to the trunk (Fig. 3). In the case where the shoulder angle is not in line with the trunk, the control scheme still assumes a fixed angle and provides support based on the elbow angle since flexion assistance can only be provided at the elbow joint.

When providing continuous support control, the system should provide assistance such that the system can move naturally with the intended arm movements. The goal is to reduce the interaction torque $\tau _i$ between user and the exosuit as much as possible such that the exosuit feels for the user as though it is an extension of the body. The high-level control scheme attempts to infer this interaction torque based on the difference between the required torque decoded from the user’s intentions through the gravity compensation control scheme and the existing assistance torque provided by the exosuit.

The assumed gravitational position-dependent torque profile for a decoded required torque $\tau _r$ is defined as a single joint model:

1 $$\begin{aligned} \tau _{r} = mgl_csin\left( \pi - \theta _e\right) \end{aligned}$$

with *m* as the combined mass of the forearm, hand, and distal part of the exosuit, $l_c$ the moment arm distance to the center of mass of the forearm and hand based on [43], *g* the acceleration of gravity, and $\theta _e$ the elbow angle relative to the shoulder irrespective of the shoulder angle $\theta _s$.

The exosuit actuators provide flexion assistance, which yields a linear relationship between pressure and assistance torque [7, 42]. Based on this model, the pressure torque relationship can be described by the following equation:

2 $$\begin{aligned} \tau _{exo} = \frac{\pi lP}{2}\left( r^2 - a^2\right) \end{aligned}$$

where $\tau _{exo}$ is the assistance torque, *l* is the actuator segment length, *P* is the actuator tube pressure, *r* is the radius of the tube, and *a* is the following:

3 $$\begin{aligned} a = \left( r + \frac{nw}{2\pi - \theta }\right) sin\left( \frac{2\pi - \theta }{2n}\right) \end{aligned}$$

with *n* as the number of segments, *w* as the distance between two successive housing chambers, and $\theta$ as the actuator angle.

The interaction torque $\tau _i$ between the required torque $\tau _r$ due to gravity and the exosuit assistance torque $\tau _{exo}$ is defined as the difference between the two torques:

4 $$\begin{aligned} \tau _{i} = \tau _{r} - \tau _{exo} \end{aligned}$$

The interaction torque $\tau _i$ should be minimal for natural arm movements and is thus set to zero for low-level control.

*Myoprocessor control* To account for various tasks and manipulated objects, an adaptive control scheme dependent on muscle activation may be beneficial. A myoprocessor control scheme based on [5] was implemented to control the exosuit based on intention decoded from muscle activation and arm position. The decoded elbow-flexion torque assumes assistance should be provided to compensate for the immediate effort detected from muscle activation. Therefore, exosuit assistance torque should be provided to compensate for the detected torque from activation of muscles. Since this activation occurs prior to physical movement onset, the exosuit is able to compensate for the predicted torque that would be provided if the exosuit was not present. Thus, the exosuit can account for the decoded torque prior to movement onset for a more adaptable and predictive control scheme. This helps reduce the physical interaction between the user and exosuit device for a more synergistic interface. The myoprocessor control scheme was implemented according to the muscle activation dynamics, a muscle force estimate, muscle kinematics, and muscle dynamics to decode a required torque about elbow.

*Muscle activation dynamics* A non-linear activation function [20] was used to determine the level of muscle activation in the biceps and triceps:

5 $$\begin{aligned} a_j(t) = \frac{e^{Au_j(t)} - 1}{e^A -1} \end{aligned}$$

where $a_j(t)$ is the activation of muscle *j* at time *t*, $u_j$ is the EMG RMS envelope at time *t*, *A* is the shape factor set to − 1 for a non-linear relationship.

*Muscle force estimate* The open-source openmuscle Python-based Hill Model implementation [45] based on work by Haeufle et al. [34] was used to determine biceps and triceps force based on EMG activation. The model assumes a three-element configuration with a contractile element, serial non-linear spring element, and parallel non-linear spring element. The estimated muscle response is based on the principle of actin and myosin cross-bridges at the sarcomere level generating muscle force according to the simplified model. Parameters were held constant based on the open-source implementation. An additional gain factor was used to scale the generated force prediction based on user preferences.

*Muscle kinematics* A simplified muscle moment arm model based on elbow angle determined from cadaveric studies [46] was used to determine the muscle moment arm for torque computation:

6 $$\begin{aligned} arm_e(\theta _e) = a_{1e} + 2a_{2e}\theta _e \end{aligned}$$

with $arm_e$ referring to both monoarticular elbow flexor (MEF) and extensor (MEE) according to Table 1 that describes the *a* constants measured.

**Table 1:** Muscle specific parameters from [46] used for moment arm calculations

Muscle	$a_{1e}$	$a_{2e}$
MEF	− 0.014	− 3.96e−3
MEE	0.025	− 2.16e−3

*Muscle dynamics* The overall muscle model combining the muscle force estimate and the muscle kinematics can be described by the following equation in which rapid flexion movements are compensated [34]:

7 $$\begin{aligned} \tau _r = l_{bi}(\theta _e)F(a_{bi}) + l_{tri}(\theta _e)F(a_{tri}) \end{aligned}$$

where $l_{bi}$ and $l_{tri}$ are the moment arms determined from MEF and MEE muscle specific parameters from Table 1 respectively and *F* refers to the muscle force generated by the respective muscle activation $a_j$.

This provides an estimate of the torque due to muscle activity and arm kinematics to determine the level of assistance needed to compensate for the required torque decoded. Similar to the gravity compensation control scheme, the required torque decoded is compensated with an exosuit assistance torque through which the interaction torque (Eq. 4) between the two should be minimized with low-level control.

#### Low-level control: optimizing system response

A simple state machine low-level controller was implemented to get a baseline understanding of the system response. The valves for controlling the exosuit were either set to an open or closed state when the interaction torque $\tau _i$ was outside of a state threshold $\tau _{state}$. To refine the control further once within the state threshold $\tau _{state}$, a PID controller was used to tune opening valve percentage $\phi _{valve}$:

8 $$\begin{aligned} \phi _{valve} = \left( \frac{\phi _{valve,max} - \phi _{valve}}{\phi _{valve,max} - \phi _{valve,min}}\right) \times 100 \end{aligned}$$

Here, $\phi _{valve}$ is the set servo angle, $\phi _{valve,max}$ is the maximum servomotor angle, and $\phi _{valve,min}$ is the corresponding minimum valve servomotor angle within the desired fully open to fully closed valve range.

A controller threshold $\tau _{c}$ is used to delineate between two separate PID controllers for the inlet and outlet valves respectively to preserve air in the system for longer operational use. Valves are controlled according to the following conditions:

9 $$ \left[ {\begin{array}{*{20}c}    {\phi _{{in}} \left( {\tau _{i} } \right)}  \\    {\phi _{{out}} \left( {\tau _{i} } \right)}  \\   \end{array} } \right] = \left\{ {\begin{array}{*{20}l}    {{\text{ }}\left[ {\begin{array}{*{20}c}    0  \\    0  \\   \end{array} } \right]} \hfill & {\left| {\tau _{i} } \right| \le \tau _{c} } \hfill & {} \hfill & {} \hfill  \\    {{\text{ }}\left[ {\begin{array}{*{20}c}    {\phi _{{in,c}} }  \\    0  \\   \end{array} } \right]} \hfill & {\tau _{{state}}  > \tau _{i}  > \tau _{c} } \hfill & {} \hfill & {} \hfill  \\    {{\text{ }}\left[ {\begin{array}{*{20}c}    0  \\    {\phi _{{out,c}} }  \\   \end{array} } \right]} \hfill & { - \tau _{{state}}  < \tau _{i}  <  - \tau _{c} } \hfill & {} \hfill & {} \hfill  \\    {{\text{ }}state_{v} } \hfill & {\left| {\tau _{i} } \right| \ge \tau _{{state}} } \hfill & {} \hfill & {} \hfill  \\   \end{array} } \right. $$

The opening valve percentage of the PID controller is denoted as $\phi _{in,c}$ and $\phi _{out,c}$ for the inlet and outlet valves respectively. The inlet valve opening percentage range was set between 50 and 80% and the outlet valve opening percentage range was set to 60–90%. This provided a sufficient range to match the decoded torque while limiting the release of air from the system to prolong operational use. When the system exceeds the defined state threshold $\tau _{state}$, the state machine control scheme takes precedent to return the system back within the state threshold for finer PID control.

### User experience: neuro-cognitive assessment

#### Study objectives

In this study, user experience during the interaction with the exosuit was evaluated through a neuro-cognitive assessment. The goal of the experiment was to determine the feasibility of detecting when the exosuit fails to decode intentions and thus violates user expectations. Participants performed a continuous tracking task while operating the exosuit in which EEG, EMG, and kinematic data were recorded to implicitly determine when the exosuit made erroneous actions. By determining when the exosuit behaves in a manner against user expectations, we can understand events in which the trust and expected usefulness, control robustness, ease-of-use, executed actions, and adaptability are affected, thereby diminishing the functional usability of the device [47]. The purpose of the study was to investigate the feasibility of decoding an expectation mismatch when operating the exosuit via the ErrP from EEG signals in a similar approach to previous human–machine interaction studies [36–40]. EMG and kinematic data were also recorded to determine if the motor response could indicate when the soft exosuit exoskeleton violated expectations. With the ability to decode when the exosuit fails to provide assistance according to expectations, the control solution can be updated to reflect user preferences. The secondary objective was to determine whether the exosuit affected control accuracy in the tracking task. For the exosuit to be usable in activities of daily living, it should not impede natural arm movements during operation.

#### Participants

Five healthy subjects between the ages of 20 and 30 (3 male and 2 female) participated in the experiment. All subjects were right-handed and used their right arm for the tracking task. All participants provided written informed consent prior to donning the exosuit. Participants were equally instructed about the experiment paradigm and given practice time to familiarize themselves with the exosuit and experiment task. Subjects were compensated 8 EUR/h for their efforts following the experiment. The study was approved by the institutional ethics review board of the Technical University of Munich under reference number 254/21 S-EB.

#### Experimental task

**Fig. 4:**
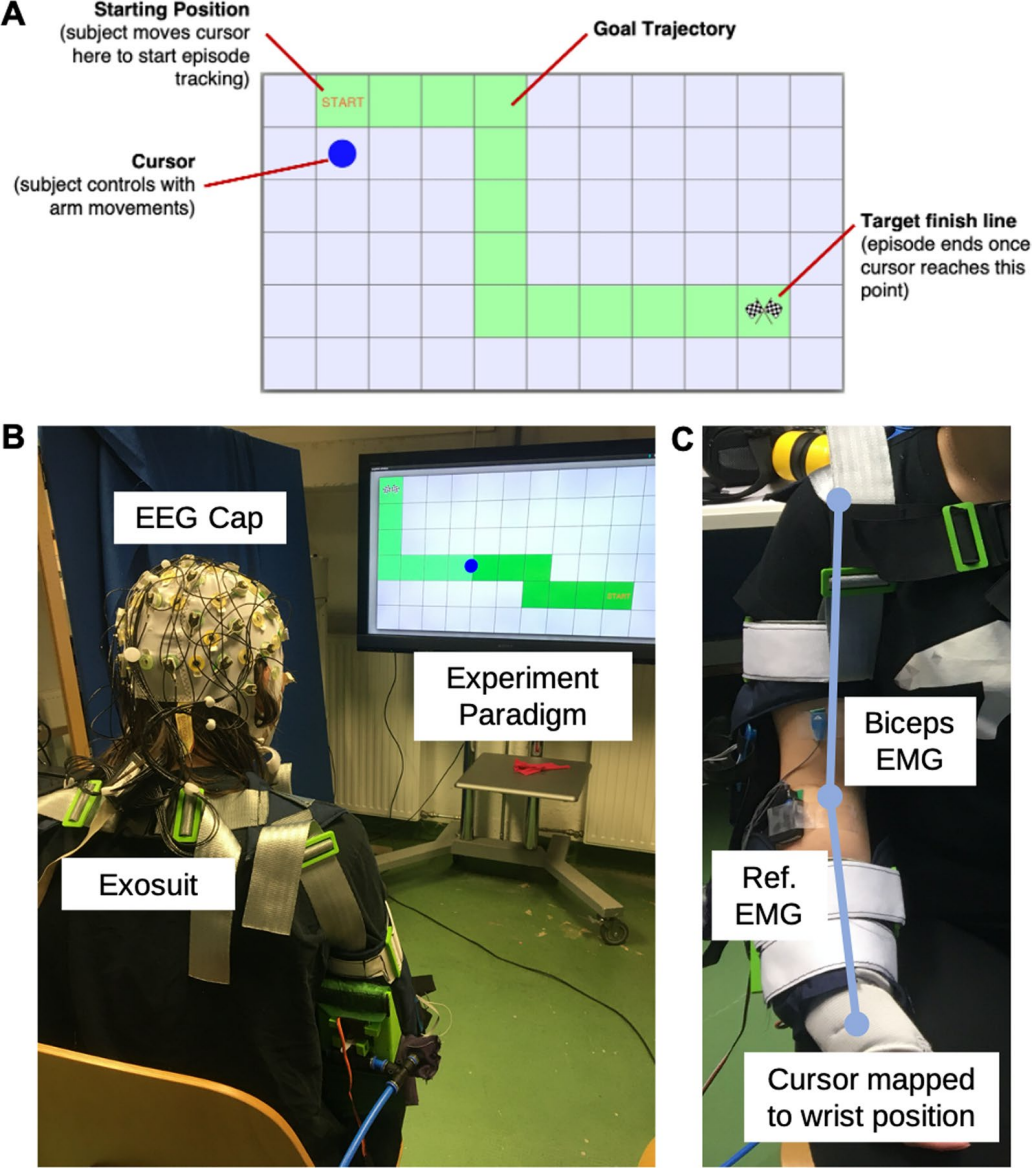
*User experience: Neuro-cognitive assessment experiment setup*. **A** Example of a single episode showing the grid layout with a goal trajectory for subjects to track cursor with corresponding arm movements. **B** Subject wearing soft exosuit sitting in a chair facing the monitor displaying the experiment. The subject is wearing an EEG cap with 32 active gel-based electrodes for measuring neural activity. In this part of the experiment, the subject is controlling the cursor through the grid trajectory. **C** The Delsys Trigno EMG system with two electrodes from the QUATTRO sensor is used to record EMG data from biceps and triceps muscles respectively. The wrist position determined from the inverse kinematics from the IMU sensors is used to map the cursor position on the screen in the task. A full range of motion was required to move the cursor throughout the entire grid, with the arm needing to reach fully flexed and extended positions from medial to lateral-right side of the body

Subjects were asked to perform a continuous tracking task while wearing the exosuit to evaluate control and accuracy. An 11 $\times$ 6 grid with a motion trajectory was displayed for subjects to follow with a cursor based on arm movements (Fig. 4A). Visually, participants saw the goal trajectory to follow and the cursor position mapped to wrist position calculated from the inverse kinematics of the exosuit (Fig. 4C). Once subjects reached the goal trajectory, a new tracking episode appeared with this process repeating until the experiment block was complete. Subjects performed the tracking task in an unassisted and assisted state with both gravity compensation and myoprocessor control schemes split up by experiment blocks. While participants performed a continuous tracking task, exosuit action events were generated by discretizing the cursor movement between grid spaces. During error blocks only, the experiment initiated purposeful control error events to determine if the errors could be detected from the brain and motor response based on the unexpected behavior. Control errors consisted of providing unnecessary assistance or releasing assistance at random steps within the grid trajectory. To avoid habituation to erroneous exosuit actions, control errors were introduced at a rate of 30% of total events in error blocks only [36], beginning at the moment when the tracked cursor moved fully into the next grid space. The introduction of artificially created errors mimicks the situation of an exosuit failing to correctly interpret the user’s intention. This provides the opportunity to posthoc validate the performance of decoding these events from the passive neural and motor response.

#### Stimuli and apparatus

*Experimental setup* Subjects were seated directly across from a LCD 42” standing monitor that displayed the experiment paradigm full-screen (Fig. 4B). Participants were fitted with the soft exosuit so that they were comfortable and understood how the device operates. Prior to using exosuit assistance, subjects were instructed to move naturally while wearing the exosuit to get a feel for how it passively moves with arm motions. We ensured subjects operated the exosuit safely and were comfortable by having them state they were ready prior to proceeding with the experiment. Throughout the experiment, subjects were instructed to relax as much as possible when performing arm motions. The air compressor was filled at the beginning of the experiment and was refilled after every third block to ensure the exosuit was properly pressurized throughout the entire experiment.

*EMG data acquisition* The Delsys Trigno EMG system with QUATTRO sensor was used for EMG recording throughout the entire experiment and exosuit control during the myoprocessor control scheme blocks. Two leads of the QUATTRO sensor were placed on the biceps and triceps respectively according to SENIAM guidelines [44] with the reference electrode on the side of the forearm (Fig. 4C). EMG sensors were wirelessly connected to the Delsys Trigno hub so that subjects were free to move their arm in space. An EMG recording ROS node integrated the EMG signal for exosuit control and synchronized it with events from the experiment. The raw EMG signal was collected at a rate of 2222 Hz and bandpass-filtered between 20 and 450 Hz. From a sliding window of 100 ms width, the Delsys transmitters computed the RMS signal at a rate of 222 Hz which was used for both control and measurement during the experiment.

*EEG data acquisition and preprocessing* EEG data was recorded using the Brain Products actiChamp system. Subjects wore an EEG cap with 32 active gel-based electrodes arranged according to an extended international 10–20 system [48] (FP1, FP2, F3, F4, F7, F8, FC1, FC2, FC5, FC6, C3, C4, T7, T8, CP5, CP6, P3, P4, P7, P8, TP9, TP10, O1, O2, Fz, Cz, Pz, EOG1, EOG2, EOG3). The mastoid electrodes TP9 and TP10 were used as the reference for all leads. Impedances for all electrodes were kept below 15 k$\Omega$ per subject and the signals were recorded with a sampling rate set at 1000 Hz. Electrooculogram (EOG1–3) signals were captured by three electrodes located on the subject’s forehead, left and right outer canthi according to Schlögl et al. [49]. The EEG amplifier was powered by a battery and connected to the recording PC located adjacent to the experiment area. The recording PC was connected via parallel port to the PC running the experiment for synchronously recording event triggers with the EEG data.

#### Experiment protocol

The overall experiment consisted of 15 blocks in total with 8 episodes per block. An episode consisted of 10–15 movement events depending on the length of the randomly generated trajectory. Each exosuit movement event was considered to be a single trial in the subsequent analysis of ERPs. The total duration of the experiment took approximately 45–60 min including breaks. A summary of the experiment protocol with the corresponding control schemes and error rates is shown in Table 2. Prior to beginning the experiment, subjects first conducted a drawing practice session in which they controlled a cursor on the screen to draw with arm movements. This ensured participants understood how the device moved with intended arm movements and the exosuit-to-cursor mapping on the screen. This practice session was repeated before beginning a block with a new control scheme. Participants were asked if they felt comfortable with the new control scheme before proceeding to the next experiment phase.

**Table 2:** Summary of the experimental protocol

Blocks	High-level control scheme	Low-level controller	Error rate (%)
1–3	Unassisted	N/A	0
4	Gravity compensation	PID	0
5–9	Gravity compensation	PID	Random(0, 0, 30, 30, 30)
10	Myoprocessor	PID	0
11–15	Myoprocessor	PID	Random(0, 0, 30, 30, 30)

#### Data analysis

*Trajectory accuracy* Trajectory accuracy was evaluated within non-error blocks. It was measured by taking the total number of correct trials out of the total number of trials of the goal trajectories. This measure for each control scheme was compared to a baseline measure of accuracy in the unassisted condition.

*EEG analysis* Data preprocessing was carried out in MATLAB using the EEGLAB toolbox [50] and used the same procedure as the study by Ehrlich and Cheng [40]. The EEG and EOG signals were filtered using a zero-phase Hamming windowed sinc FIR bandpass filter between 1 and 20 Hz. Contaminated channels were determined using kurtosis with a 5% threshold and correspondingly interpolated. Eye blinks were corrected via the EOG signals based on Schlögl et al. [49]. All electrodes were re-referenced to a common average reference of all channels to further reduce noise in the signal.

Single trial ERPs were epoched time-locked to the onset of movement beginning when the cursor was fully contained within a grid space at which point the grid space changed color to a darker shade of green for a correct step (Fig. 4B). Epochs began at the event-onset and ended one second post step-onset to account for the varying speed at which subjects moved the cursor. In error blocks within the respective control scheme, trials were grouped based on whether or not a control error was present. Pearson correlation across the negative deflection from 300 to 400 ms post-event onset was determined between trials in different control schemes for a similarity measure.

For classification of errors, temporal features were selected by first downsampling the epochs to 125 Hz and selecting a sub-set of channels of interest, namely Fz, F3, FC1, C3, Cz, C4, T8, FC6, FC2, F4, and F8 for approximately 200 features per event. Channels were selected based on feature discriminability and expected spatial location of neural response based on insights provided by earlier works on decoding ErrPs [36, 39]. Dimensionality reduction was then performed to extract latent features through principal component analysis (PCA) to increase the separability of features based on variance. The temporal features were evaluated using a Fisher score analysis to determine the discriminative power. Subsequently, a regularized linear discriminant analysis (rLDA) classifier based on [51] was used to discriminate events based on the labeled overall groupings of control error and non-error trials. The regularization aims to minimize the covariance estimation error by penalizing small and large scalings of the hyperplane discriminating the feature space. This classifier was chosen based on success in previous BCI works in the decoding of ErrPs [39, 40]. The classification problem between control error trials and non-error trials was validated with a tenfold cross validation. For each subject, trials were randomly split into tenfolds with ninefolds used for model calibration and the remaining fold for testing. This procedure was repeated ten times in total for an estimate on how well a subject-specific decoder would perform on unseen data within a single session. Classification results per subject are reported as the average percentage of correctly classified trials across all folds. This provides a subject-specific decoder accuracy of detecting unexpected exosuit actions based on user perceptions.

*EMG analysis* The preprocessed RMS signal was used to determine EMG muscle activation of both biceps and triceps muscles. The signal was epoched and grouped using the same method as the EEG analysis. Epochs began 0.2 s before event onset and continued for 1.5 s after the event beginning. Temporal RMS features from biceps and triceps channels were extracted and subsequently reduced dimensionally using PCA. Classes were grouped based on error and non-error trials in the error-blocks. The classification problem was handled identically to the EEG classification analysis with a rLDA classifier and tenfold cross validation to report overall EMG model accuracy for correctly predicting error events based on EMG activity.

*Kinematic analysis* The angular velocity of the elbow joint was also measured to determine when subjects experienced rapid changes in elbow flexion or extension. Trials were epoched according to the same procedure as the EEG and EMG data analysis with trials consisting of control errors and non-errors. The raw elbow angular velocity was reduced dimensionally using PCA. The identical classification procedure as the EEG and EMG analysis (rLDA classifier and tenfold cross validation) was used to classify error trials based on kinematic data.

#### Statistics

**Fig. 5:**
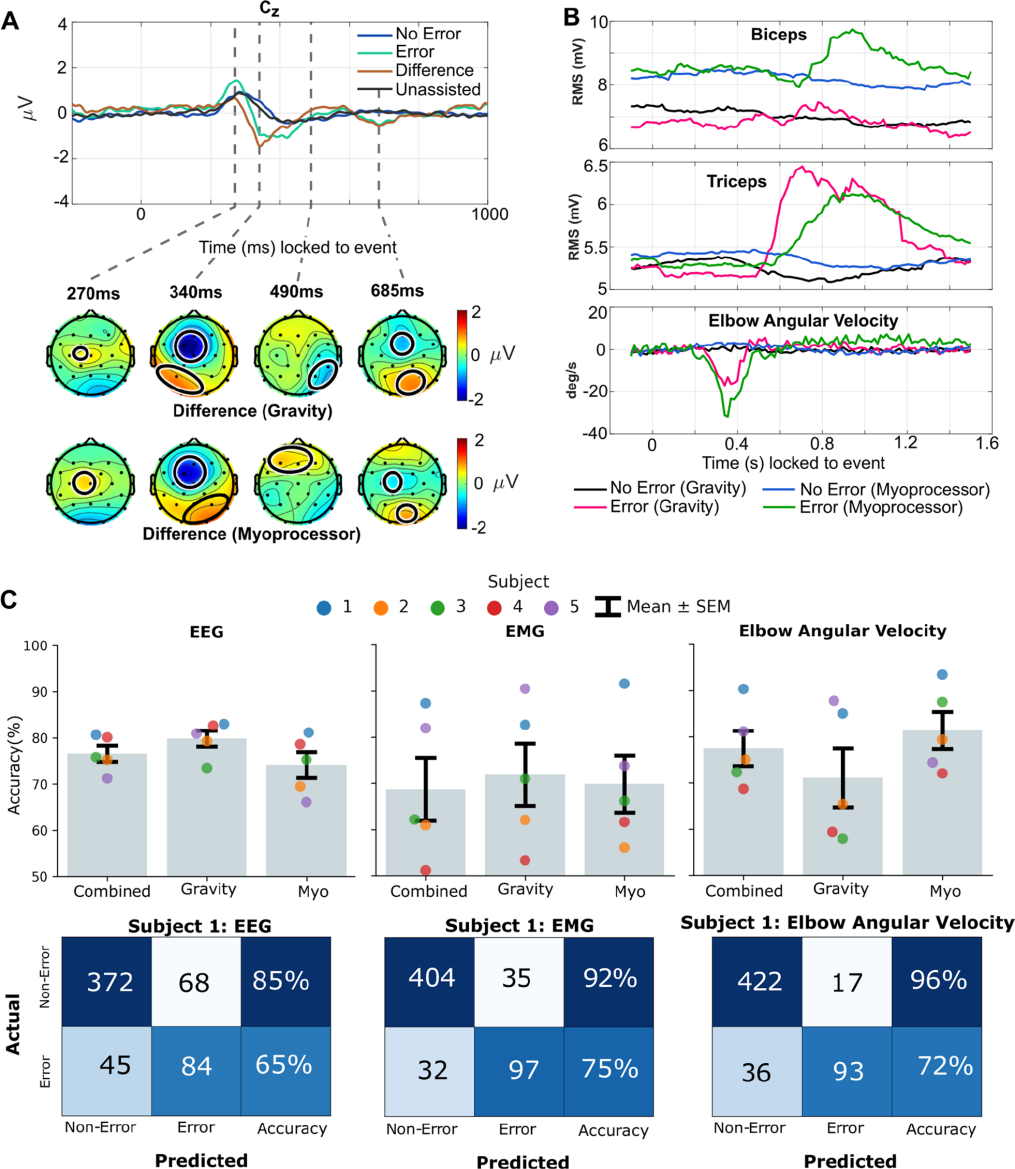
*Neuro-cognitive assessment results* **A** Grand average ERP response in channel Cz from all subjects combining gravity and myoprocessor control schemes for error vs. non-error trials in error blocks. Difference represents the difference between error and non-error trials. The response during the unassisted blocks is shown as a reference. Topographic visualizations of the difference between error and non-error trials in error blocks for gravity and myoprocessor control schemes respectively are shown below the ERP time-course plot. Regions outlined show areas of significant difference from Tukey HSD tests with $p<0.05$. **B** Grand average motor response (biceps, triceps, and elbow angular velocity) from all subjects grouped by control scheme for error vs. non-error trials in error blocks. **C** Classification results from user experience neuro-cognitive assessment study. Trained rLDA models based on signals from EEG, EMG, and elbow angular velocity were used to classify control error trials during error blocks. The gravity and myoprocessor (Myo) control methods were compared to determine if accuracy was affected based on the control measure. Additionally, a combined accuracy for both control methods was determined per model. Error bars indicate mean ± SEM across subjects per model and control scheme. Representative confusion matrices for the combined control scheme models from subject 1 are depicted beneath the group results

Tukey honestly significant difference (HSD) tests were used to determine EEG channel regions of significant difference between error and non-error trials. Additionally, a Tukey HSD test was used to cross-compare the unassisted, gravity, and myoprocessor control conditions with respect to trajectory accuracy. Group decoding accuracy distributions were tested with the Lilliefors test to determine normality. Paired t-tests were used to compare decoding accuracy between control schemes across the different modalities. A power analysis was conducted to determine the minimum number of subjects needed for significant difference. Statistical testing was conducted using Python 3.7 with SciPy and Statsmodels packages. Error bars indicate mean ± standard error of the mean (SEM) in Fig. 5.

## Results

### Trajectory accuracy

Overall, subjects were able to perform the tracking task while operating the exosuit. The physical interface did not restrict their ability to achieve a high accuracy in the unassisted or assisted conditions (Table 3). As a result, the exosuit control system did not cause a significant performance reduction with regard to precision of movements and control with no significant difference in tracking performance (Tukey HSD: $p>0.05$ for all comparisons).

**Table 3:** Overall subject trajectory accuracy in tracking task during non-error blocks only

Control scheme	Accuracy ± $\sigma _{acc}$ (%)
Unassisted	98.50 ± 1.94
Gravity	98.52 ± 1.30
Myoprocessor	97.56 ± 2.20

### Event-related potential and motor response

Grand average ERP responses from channel Cz for both control schemes are depicted in Fig. 5A. Based on the grand average ERP response, both control schemes had similar ERP waveforms (Pearson correlation: 99.0% and 82.5% correlated across the negative deflection for non-error and error trials). Therefore, there is no statistical difference between error-related negativity (ERN) despite the gravity control scheme having a slightly more prominent peak (t = 340 ms). In response to control errors, subjects elicited ErrPs characterized by the strong ERN at approximately 340 ms post event triggered onset. In particular, subject 4 elicited the strongest ERN peaking at − 4$\mu V$ in channel Cz (Appendix Fig. 6). Prior to the ERN, subjects 1 and 5 elicited a positive peak at approximately 270 ms in error trials resulting in a slightly positive peak in the grand average ERP response. The time delay in ERN of the ERP is due to the lag in device movement occurring at approximately 200 ms post event onset (see Elbow angular velocity in Fig. 5B). A more sustained positive deflection occurred between 400 and 600 ms post event onset that was particularly evident for subjects 3, 4, and 5. A final negative deflection is evident at approximately 685 ms with subjects 1 and 5 driving this response in the grand average results. Despite some differences in ERP waveforms, all subjects elicited the characteristic ERN of the ErrP response similarly found in previous works [36–40]. Lastly, the grand average response between the unassisted and non-error trials in error blocks share an almost identical waveform suggesting that when assistance is provided as intended, the control support does not alter the neural response to exosuit-cursor movement events.

Topographic visualizations for the grand average difference between error trials and non-error trials for gravity and myoproccesor blocks are shown in Fig. 5A. The topographical visualizations highlight the pronounced ERN in the fronto-central channels for both control schemes with a grand average peak of − 2 $\mu$V. Significant difference regions (Tukey HSD: $p<0.05$) between error and non-error trials are outlined. At the four time points of interest, most of the significant regions appear to be in the central channels consistent with previous ErrP findings [36–40].

Grand average RMS muscle activations and angular velocity profiles grouped by error and non-error trials in error blocks are shown in Fig. 5B. A clear difference between error trials and non-error trials is evident for both biceps and triceps activation, indicating that subjects needed to respond to the errors to retain control of the exosuit. Additionally, there is a noticeable elbow angular velocity peak at 350 ms post event onset. This indicates that the errors caused a rapid elbow flexion as intended with corresponding muscle response beginning approximately 0.5 s after the error trigger that successfully stabilizes the angular velocity.

When comparing the control schemes, the gravity compensation control results in overall lower muscle activation as compared to the myoprocessor control scheme. Additionally, with lower levels of muscle activation, the response in error trials for the gravity scheme are less pronounced for the biceps. The triceps activation appears to show a similar response for both control schemes with the average triceps response acting slightly quicker for the gravity control. Elbow angular velocity peaks occur for both control schemes at the same time with the myoprocessor peak being more prominent (31°/s) than the gravity compensation peak (18°/s).

### Classification of control errors

EEG classification models were trained for the combined error trials of both control schemes and also the respective independent control scheme groups. The classification accuracy of decoding error trials in the error blocks are shown in Fig. 5C. A grand average accuracy of 76.63 ± 1.73% was achieved for the combined control schemes indicating that the model is able to classify control error trials consistent with previous human–machine interaction ErrP studies [39, 40]. Within control scheme, a grand average accuracy of 79.89 ± 1.73% and 74.15 ± 2.80% was achieved for the gravity and myoprocessor control schemes respectively. There is no statistical difference in decoding accuracy between control schemes (paired t-test gravity vs. myoprocessor; EEG: $p=0.243$). A power analysis revealed that an additional 5 subjects with identical deocding accuracies (10 subjects total) would yield significantly different results between the gravity and myoprocessor control schemes (paired t-test gravity vs. myoprocessor; EEG: $p=0.019$).

Subject specific models from EMG and elbow angular velocity signals were trained independently and as a combined grand average to classify error trials based on the respective motor response. Classification accuracy for both model sets is shown in Fig. 5C. Overall grand average decoding accuracy for the combined control schemes was 68.73 ± 6.83% and 77.52 ± 3.79% for the EMG and kinematic models respectively. In contrast to the EEG classification results, the EMG and kinematics results vary more significantly by subject. Within the gravity compensation control scheme, decoding accuracy was 71.92 ± 6.71% and 71.13 ± 6.38% for the EMG and kinematic models respectively. Myoprocessor decoding accuracy achieved 69.88 ± 6.15% and 81.38 ± 4.01% for the EMG and kinematic models respectively. There is no significant difference in decoding accuracy between control schemes from the motor response (paired t-test gravity vs. myoprocessor; EMG: $p=0.694$ and kinematics: $p=0.211$).

## Discussion

### High-level control: decoding user intention

For an assistive device to be accepted by the user, it should be intuitive to operate and provide support in line with user expectations [47]. To predict movement intention with a gravity compensation scheme, we assume that the upper arm is in line with the wearer’s trunk. However, in reality the user is free to move the upper arm, which subsequently means that the gravity compensation scheme takes the relative elbow angle into account. Intuitively this makes more sense for the wearer because it means that as the elbow is flexed, assistance will be provided with respect to the elbow angle. To further align with user intentions, the full arm kinematics should be considered. However, with the current exosuit construction, we are only able to provide assistance about the elbow and cannot fully actuate according to a user’s full range of motion. Therefore, to simplify the ease-of-control, we set the exosuit to provide assistance based on elbow angle irrespective of where the upper arm is in space.

Compared to a myoprocessor based scheme for decoding intention, a kinematics-based solution may be sufficient and streamline the setup process as it does not require a lengthy setup with manual EMG sensor placement and calibration. Instead, the exosuit can be manually set for different object masses based on the user’s desired task. While an operator is likely to interact with varying sized objects, the exosuit in this configuration, while not adaptive, can provide the wearer with an average level of support based on the set assistance level. Another option is to potentially integrate the exosuit with a supplementary smart glove to detect an approximate grip or hold of an object for an adaptive force prediction [52]. This would allow the gravity compensation scheme to adapt the level of assistance based on the predicted mass without the need for pre-determining object mass or introducing costly EMG sensors. Overall, while the gravity compensation control scheme has some limitations, it provides an intuitive method to control the exosuit for a variety of different tasks.

Myoprocessor intention detection, on the other hand, offers the adaptability to account for various interactions with the environment based on muscle activity. Additionally, neuromuscular signals can be detected prior to movement, thereby allowing the control loop to predict movement intentions and provide assistance accordingly [20, 21]. Taking these two factors into consideration, an EMG-based control scheme may be a superior control option compared to a purely kinematics-based approach [6]. To increase the usability of the system, our approach was to simplify the setup as much as possible. Rather than conducting a lengthy calibration procedure for each participant, we held the myoprocessor pipeline and gain factor constant regardless of muscle activity level. While the torque provided varied between participants due to imprecise sensor placement and baseline muscle activity level, participants were able to control the exosuit with minimal training. Since EMG signals are non-stationary overtime, an adaptive gain factor would likely be a superior option to account for long-term use [53]. A high-density EMG array with sleeve design may also provide additional information to reduce setup time and increase the usability of the system [54]. While there are performance advantages of myoprocessor control, depending on the scenario, a gravity compensation control may be more robust with the current exosuit construction.

With both control methods, an initial interaction torque is required to initiate exosuit control. In a fully flexed position, this requires some initial force generation by the user to backdrive the exosuit through actuation, which may limit its application in rehabilitation settings. Additionally, this has the potential to diminish the cognitive interaction with the device as it should not impede natural arm movements. To address this, our group is investigating using sensors at the interface between the exosuit and user to support passive and active movement, as well as support resistance training for rehabilitation [55]. In addition, a predictive model-based low-level controller has the potential to reduce lag in the system, thereby minimizing the interaction torque for the user. With that said, as different control methods are introduced, the user experience with the exosuit should be considered to enhance device acceptance.

### User experience: neuro-cognitive assessment

Our neuro-cognitive assessment challenged participants in a tracking task to control the exosuit within a target trajectory. Participants were able to learn the mapping between the exosuit and cursor movement, as well as adapt to the two control schemes quickly for high accuracy scores. Intentional control errors introduced diminished subject accuracy and were observable in the user’s EEG and EMG, as well as kinematic activity from rapid arm movement changes. Based on the EEG results, we noticed that subjects elicited ErrPs characterized by a strong ERN indicating an expectation mismatch [56]. Overall subject responses varied in magnitude and waveform shape, but all shared a deflection that can be associated with the ERN for control error trials. Differences in ERN magnitude and ERP response may have been boosted by various ways of interacting with the system and task, such as speed variation or muscle co-contraction. We noticed that some subjects attempted to avoid errors by either moving through portions of the experiment extremely fast (e.g. when the cursor was against a border because it was easy to avoid making mistakes) or by co-contracting to avoid the severity of the error. While these few subjects learned to bypass the control errors, they still elicited responses indicative of an expectation mismatch via an ErrP and increase in motor response activity.

Trained classification models were able to decode control error responses with a grand average accuracy of 76.63 ± 1.73% from EEG, 68.73 ± 6.83% from EMG, and 77.52 ± 3.79% from elbow angular velocity, which is consistent with previous ErrP studies [36–41] ranging in decoding accuracies between 50 and 80%. In these works, ErrP decoding accuracy varied based on classification method, interaction agent modality (e.g. robot or screen-based), and closed versus open-loop control scenarios. In closed loop scenarios, it was demonstrated that despite relatively low ErrP decoding accuracies with respect to chance level, the detection of errors could be used to successfully update robot interaction strategies toward an optimal solution for the user and robot. For a slow reinforcement learning update, a high decoding accuracy is not required for the robot and human to co-adapt [57]. Therefore, understanding when the exosuit fails to meet expectations can elucidate instances in which the exosuit should update its control policy.

Aside from closed loop scenarios, a neuro-cognitive assessment can help understand which control methods are in line with user preferences. In our study, while errors were introduced artificially, they have the potential to provide information about the underlying intentional control mechanism based on decoding accuracy performance. The decoding accuracy can be seen as a proxy for the reliability of the device according to user expectations based on the decoder’s ability to dissociate purposeful control errors from normal device operation. While we were unable to see statistically significant differences in decoding accuracies between the two control methods due to the low sample size, we demonstrate the feasibility of using this method to assess the overall cognitive response to the intentional control schemes. A power analysis indicated that a minimum of 10 subjects would be required to determine significant difference between control schemes. Interestingly, within control schemes, the EMG decoding accuracy positively correlates with the EEG accuracy, with the elbow angular velocity decoding accuracy being inversely proportional to both the EEG and EMG results. A lower decoding performance from the elbow angular velocity in the gravity compensation control may be due to the less prominent deflection at the onset of the control errors. Decoding accuracy based on the motor response varied between subjects, with trained models from subjects 1 and 5 achieving a higher overall accuracy. Differences in decoding performance from the motor response can be attributed to the various ways of interacting with the task.

A limitation of our study is the successive order of control methods that subjects performed the tracking task. It is likely that over time, subjects could have gained familiarity with the task, as well as experienced muscular and cognitive fatigue that could have impacted the results. Additionally, the tracking task does not guarantee the dissociation between visual and tactile stimuli. A limitation of our study is that we do not dissociate the visual and tactile stimuli. While we were able to see a significant difference between control error trials and non-error trials, it remains unclear if users responded to the visual cursor movement rather than the physical control response. Subjects were able to learn the exosuit-cursor mapping quickly, but when moving fast through the tracking paradigm, it is possible that the visual cursor movement may have induced an expectation mismatch rather than the physical exosuit actuation. While with the given study, an exact description of the causal origin of the observed ErrP responses (caused by visual or proprioceptive sensory mismatch) was not possible, the motor response, in addition to decoding performance from the motor response, corroborates our findings that subjects cognitively realized the physical control error. A future study should have a design that allows the isolated examination of ErrPs evoked by visual and somatosensory perceptual modalities. Our findings suggest that through decoding unexpected exosuit actions, we can determine when the exosuit fails to perform actions in line with user expectations. This has the potential to inform us in which situations the control can be modified for a sense of embodiment in which the device operates as an extension of the body. When applied in more realistic scenarios without a visual tracking paradigm and in a closed-loop scheme, the neuro-cognitive response, error-related muscle activity, and error-related kinematic activity can inform decisions on how to update exosuit control to enhance the cognitive human–machine interface.

## Conclusion

We developed a soft pneumatic elbow exosuit capable of providing elbow flexion assistance in accordance with user intentions. The soft lightweight design provides a suitable interface for the wearer that does not restrict arm motions. Gravity compensation and myoprocessor control schemes based on existing state-of-the-art methods were implemented and validated with the exosuit construction for an intuitive control interface. We demonstrated the feasibility of decoding unexpected exosuit actions via a continuous paradigm through the wearer’s neuro-cognitive and motor response. This work addresses current limitations in wearable robotics by evaluating device performance with respect to the user’s cognitive response to determine when the exosuit fails to perform actions in line with user expectations.

## Figures and Tables

**Fig. 6:**
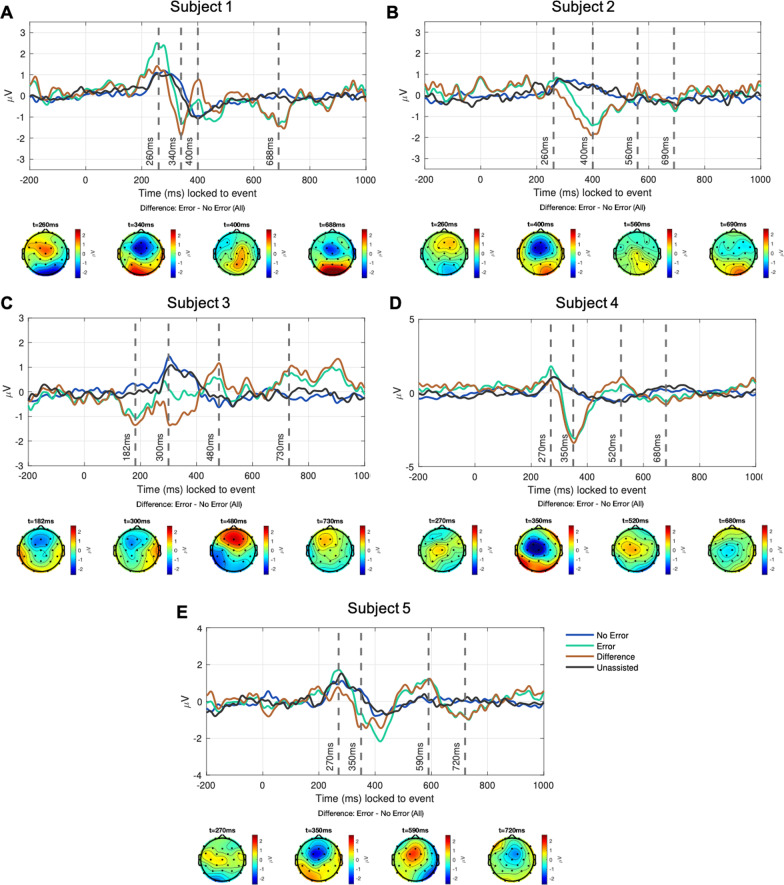
*Individual subject EEG results from the neuro-cognitive assessment* **A**–**E** Average ERP response in channel Cz per subject combining gravity and myoprocessor control schemes for error vs. non-error trials in error blocks. Difference represents the difference between error and non-error trials. The response during the unassisted blocks is shown as a reference. Topographic visualizations of the difference between error and non-error trials at time points of interest in error blocks combining gravity and myoprocessor control schemes are shown below the ERP plots

## Data Availability

The datasets used and/or analysed during the current study are available from the corresponding author on reasonable request.

## Appendix

See Fig. 6.
